# Regulation of Human Neurotropic JC Virus Replication by Alternative Splicing Factor SF2/ASF in Glial Cells

**DOI:** 10.1371/journal.pone.0014630

**Published:** 2011-01-31

**Authors:** Ilker Kudret Sariyer, Kamel Khalili

**Affiliations:** Department of Neuroscience and Center for Neurovirology, Temple University School of Medicine, Philadelphia, Pennsylvania, United States of America; University of Nebraska - Lincoln, United States of America

## Abstract

**Background:**

The human neurotropic virus, JC virus (JCV), is the etiologic agent of the fatal demyelinating disease of the central nervous system, Progressive Multifocal Leukoencephlopathy (PML) that is seen primarily in immunodeficient individuals. Productive infection of JCV occurs only in glial cells, and this restriction is, to a great extent, due to the activation of the viral promoter that has cell type-specific characteristics. Earlier studies led to the hypothesis that glial-specific activation of the JCV promoter is mediated through positive and negative transcription factors that control reactivation of the JCV genome under normal physiological conditions and suppress its activation in non-glial cells.

**Methodololgy/Principal Findings:**

Using a variety of virological and molecular biological approaches, we demonstrate that the alternative splicing factor SF2/ASF has the capacity to exert a negative effect on transcription of the JCV promoter in glial cells through direct association with a specific DNA sequence within the viral enhancer/promoter region. Our results show that down-regulation of SF2/ASF in fetal and adult glial cells increases the level of JCV gene expression and its replication indicating that negative regulation of the JCV promoter by SF2/ASF may control reactivation of JCV replication in brain.

**Conclusions/Significance:**

Our results establish a new regulatory role for SF2/ASF in controlling gene expression at the transcriptional level.

## Introduction

JCV is a human polyomavirus that infects greater than 80% of the human population during childhood [1], [2]. Replication of the neurotropic strain of JCV in glial cells causes the fatal demyelinating disease of the central nervous system, progressive multifocal leukoencephalopathy (PML), which is seen in patients with underlying immunocompromised conditions, notably among AIDS patients [3], [4], [5], [6]. Recently, several cases of PML have been reported in patients under treatment with immunomodulatory drugs including Natalizumab, Rituximab, and Efalizumab, indicating that alterations in immune status may lead to reactivation of latent and/or passing virus in human brain [7], [8], [9], [10]. Like other polyomaviruses, the JCV genome is composed of double-stranded circular DNA of approximately 5 kb in size with a bi-directional non-coding control region that is located between the early and late coding sequences. The early coding region is responsible for expression of large T-antigen, small t-antigen, and a group of T' proteins, all of which are produced upon alternative splicing of a specific primary transcript [11]. Similarly, alternative splicing of the late transcript results in production of the viral capsid proteins VP1, VP2, and VP3 all of which are important for completion of the viral lytic cycle and formation of viral particles. Processing of both early and late primary transcripts requires participation of splicing factors including SF2/ASF, an ubiquitous factor which plays a pivotal role in alternative and constitutive splicing of precursor mRNAs of mammalian cells [12], [13], [14], [15], [16]. Of particular interest, the notion that SF2/ASF was first discovered as a cell type-specific regulator of another well-studied member of the polyomavirus family, SV40, based on its ability to modulate splicing of the viral early gene, thus effecting the expression of large T-antigen and small t-antigen expression at the post transcriptional level [17], [18]. The non-coding control region of the neurotropic strain of JCV, Mad-1, is composed of two 98 bp tandem repeats that have cell type-specific characteristics and its activation primarily occurs in glial cells such as oligodendrocytes and astrocytes [3], [4]. Earlier results from our laboratory and others led to the identification of several constitutive and inducible cellular factors with the ability to positively and negatively control JCV gene transcription [19], [20]. These observations led us to postulate that transcription of the JCV promoter is controlled by a group of transcription factors that universally silence expression of the viral genome under normal conditions and the level of their expression and/or activities are changed under certain physiological conditions such as immunosuppression, thus providing an opportunity for the virus to replicate in the permissive cells of the CNS. Here, we examined the possible effect of SF2/ASF on the regulation of JCV gene expression and viral replication in glial cells. Our results show that while SF2/ASF plays a role, similar to that seen in SV40, in splicing viral transcripts, it has a profound impact on transcription of the viral genome and replication of JCV in glial cells. Our results show that overexpression of SF2/ASF suppresses JCV gene transcription in human glial cells. Accordingly, suppression of SF2/ASF enhances the level of viral replication in astrocytic cells. These observations provide the first evidence for regulation of a promoter activity by the splicing factor, SF2/ASF, and shed new light onto regulation of JCV gene transcription and replication.

## Results

### SF2/ASF suppresses replication of JCV in glial cells

**Figure 1:**
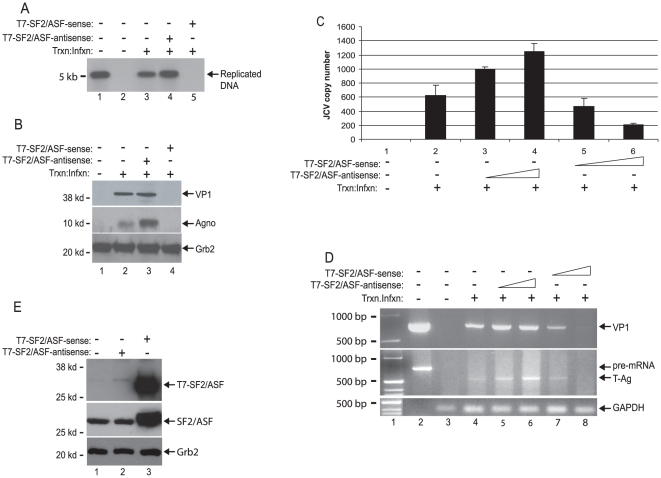
Overexpression of SF2 inhibits JCV propagation in PHFA cells. A. Southern blot analyses of JCV-infected PHFA cells. In lane 1, 2 ng of lineralized Mad-1 genome was used as positive control. In lane 2, DNA samples from uninfected cells were loaded as negative control. B. Western blot analyzes of whole cell extracts prepared in parallel to DNA samples in panel A, using specific antibodies against VP1 and agnoprotein. In lane 1, whole cell extracts from uninfected cells was loaded as negative control. Western blot analyzes of same extracts with anti-Grb2 antibody was used as loading control. C. Q-PCR analyses of the viral particles in the JCV infected-cells culture medium. D. RT-PCR analyses of JCV early (T-Ag) and late (VP1) gene products in JCV infected PHFA cells. In lane 1, Kb ladder was loaded as molecular weight marker. In lane 2, JCV Mad-1 genome was used as a positive control. E. Western blot analyzes of endogenous (SF2/ASF) and overexpressed (T7-SF2/ASF) levels of SF2/ASF in PHFA cells. Grb2 was probed in the same membranes as loading control.

To investigate the effect of SF2/ASF on replication of JCV, primary human fetal astrocytes, PHFA, were transfected with JCV Mad-1 DNA, either alone or together with a plasmid expressing SF2/ASF in sense or antisense orientation. Overexpression of SF2/ASF had a negative effect on replication of JCV DNA (Fig. 1A) and production of the viral proteins, VP1 and agnoprotein (Fig. 1B). In the antisense orientation, expression of SF2/ASF showed no inhibitory effect, suggesting that the overexpression of SF2/ASF, as shown in Fig. 1E, is required for the observed suppression. Expression of SF2/ASF in PHFA also decreased the copy number of the virus during the course of infection, indicating that the decrease in viral gene expression by SF2/ASF has an impact on the viral lytic cycle and its propagation (Fig. 1C). In cells expressing SF2/ASF in the antisense orientation, more virus was detected in the culture media. Examination of viral transcripts by RT-PCR further established the inhibitory effect of SF2/ASF on expression of the JCV early and late genome (Fig. 1D).

### Effect of SF2/ASF on splicing of the JCV transcript

**Figure 2:**
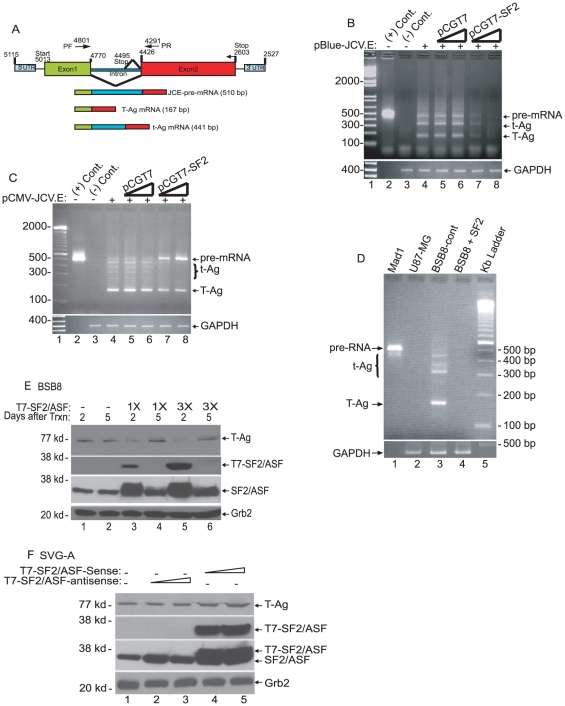
Effect of SF2/ASF on JCV early gene splicing in glial cells. A. Schematic structure of JCV early region unspliced and spliced RNAs and, the size of the expected amplification products with a primer set (PF and PR), used for the amplification of JCV gene products in panels B, C, and D. B. SF2/ASF inhibits expression of JCV early region driven by its own promoter. pBlueScript KS JCV early reporting JCV early region under JCV promoter (pBlue-JCV.E), was transiently transfected into PHFA cells in the presence or absence of an SF2/ASF expression plasmid. Total RNA was extracted and used for cDNA synthesis by reverse transcriptase reaction. JCV-early region gene products (pre-mRNA, t-Ag and T-Ag) were amplified and separated on a 3% agarose gel and stained with ethidium bromide. Lane 2 was pBlue-JCV.E plasmid DNA amplified as positive control. Lane 3 was untransfected PHFA cell extracts used as a negative control. All the bands reflecting the amplification products were separately cut from the gels, purified and RNA identities were confirmed by sequencing. GAPDH was also amplified in the same cDNA samples as input control. C. SF2/ASF inhibits splicing of JCV early region driven by CMV promoter. PHFA cells were transiently-transfected with pCMV-JCV.E in the presence or absence of an SF2/ASF expression plasmid. Total RNA was extracted and processed for reverse transcriptase reaction as described in panel B. In lane 2, pCMV-JCV.Early plasmid DNA was amplified and loaded as a positive control of primary transcript. Lane 3 was un-transfected PHFA cell extracts and was used as negative control of amplification. D. BSB8 cells were transfected with either vector alone (lane 3) or with SF2/ASF (lane 4) expression plasmids. Expression of JCV-Early gene products were analyzed by RT-PCR as described in panels B and C. U-87 MG cells (lane 2) were also processed as lanes 3 and 4, and were used as negative control of amplification. E. Over-expression of SF2/ASF inhibits T-Ag expression in BSB8 cells. BSB8 cells were plated in 6-well dishes and transfected with SF2/ASF expression plasmid in increasing concentrations (1X and 3X). Whole cell extracts were prepared at 2nd and 5th days post-transfection and processed by Western blotting using specific antibodies against T-Ag, SF2/ASF, and T7-SF2/ASF. Grb2 was probed as a loading control after stripping of the same primary membranes used for Large T antigen and T7-tagged SF2/ASF protein detections. F. SF2/ASF shows no inhibitory effect on SV40 Large T antigen expression in SVG-A cells. SVG A cells were plated in 6-well plates and transfected either with SF2/ASF-sense or with SF2/ASF-antisense expression plasmids. T-Ag expression was detected by Western blotting. Expression of SF2/ASF and Grb2 was detected in the same blots by using SF2/ASF-specific, T7-specific and Grb2-specific antibodies.

To further demonstrate the effect of SF2/ASF on splicing of the JCV transcript, a plasmid containing the JCV early promoter expressing the early transcripts was introduced into PHFA along with a plasmid expressing SF2/ASF (pCGT7-SF2) or the empty vector (pCGT7). RNA analysis was carried out by RT-PCR using primers that amplify alternatively spliced forms of JCV early transcripts (Fig. 2A). Interestingly, results showed that ectopic expression of SF2/ASF significantly diminished the level of the viral transcript, suggesting that SF2/ASF may have a negative effect on transcription of the JCV promoter (Fig. 2B). To further investigate the specificity of this observation, PHFA were transfected with a plasmid containing the CMV promoter expressing the JCV early transcript. As shown in Fig. 2C, expression of SF2/ASF had no effect on transcription of the CMV promoter expressing and that JCV early RNA was synthesized and in cells with overexpression of SF2, unspliced viral RNAs were accumulated and splicing of the primary transcripts for production of small t-antigen RNA was severely suppressed. These observations suggest that similar to its effect on splicing of SV40 [18], overexpressed SF2/ASF can also impair JCV early RNA splicing.

The unanticipated observation of the suppression of JCV promoter activity by SF2/ASF in transfected cells prompted us to determine whether transcription of the integrated copy of the JCV genome in cellular chromosomes can be influenced by SF2/ASF. To this end, we utilized a transgenic mouse cell line containing the JCV early promoter expressing the early genome. Results from RNA analysis by RT-PCR showed that ectopic expression of SF2/ASF severely suppresses production of JCV RNA but not control GAPDH transcripts (Fig. 2D). Examination of early gene expression at the protein level corroborated results from RNA analysis showing that production of SF2/ASF inhibits accumulation of the viral early protein, T-antigen, in the cells (Fig. 2E). Again, this observation was specific to JCV gene expression as evaluation of SV40 early protein production in SVGA, which contains an integrated copy of the SV40 early region, showed no effect of SF2/ASF on SV40 T-antigen expression (Fig. 2F).

### Inhibition of JCV promoter activity by SF2/ASF

**Figure 3:**
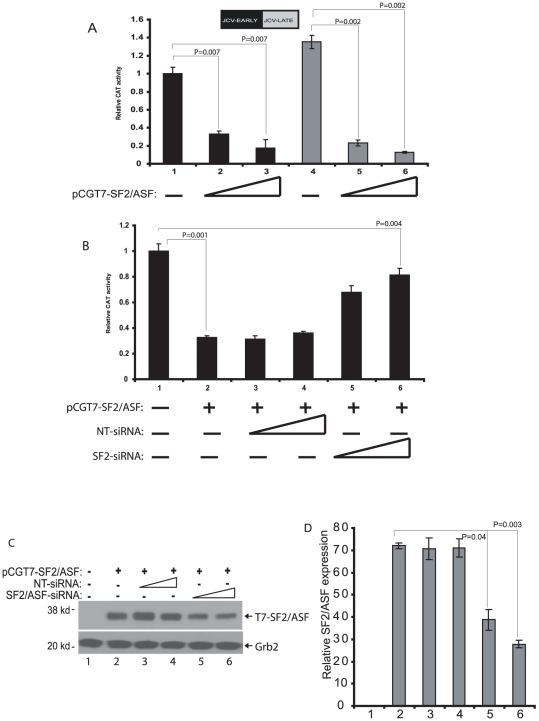
SF2/ASF inhibits JCV-early (JCV_E_) and –late (JCV_L_) transcription in PHFA cells. A. JCV_E_ (black bars) or JCV_L_ (grey bars) reporter plasmids were transiently transfected into PHFA cells either alone or in combination with an SF2/ASF expression plasmid. B. SF2/ASF-mediated inhibition of JCV-early transcription can be rescued by a specific siRNA against SF2/ASF. JCV_E_ reporter plasmid was transiently transfected into PHFA cells either alone or in combination with an SF2/ASF expression plasmid (lanes 2-6). At 12 h post-transfection, cells were transfected either with a non-targeting siRNA or with a siRNA specific to SF2/ASF. C. Western blot analyses of the same extracts used for reporter assays in panel B, using specific antibodies to T7-SF2/ASF and Grab2. D. Band intensities of SF2/ASF expression from panel E were quantified and showed as bar graph. The Student's t-test was performed to calculate “P” values in panels A, B, and D.

In the next series of experiments we measured the effect of SF2/ASF on transcription of JCV early and late promoters. Results from a series of co-transfection experiments showed that overexpression of SF2/ASF in glial cells suppresses the activities of both the JCV early and late promoters (Fig. 3A), whereas inhibition of SF2/ASF abrogates JCV promoter activity inhibition by SF2/ASF (Fig. 3B). The specificity of this observation was tested with transcription assay using E2F-1, and BKV promoter sequences. Notably, under similar conditions, ectopic expression of SF2/ASF exhibited no inhibitory impact on the transcription of E2F-1, and BKV-early and –late promoter activities (Fig. S1), pointing the specificity of our observation on JCV gene transcription.

**Figure 4:**
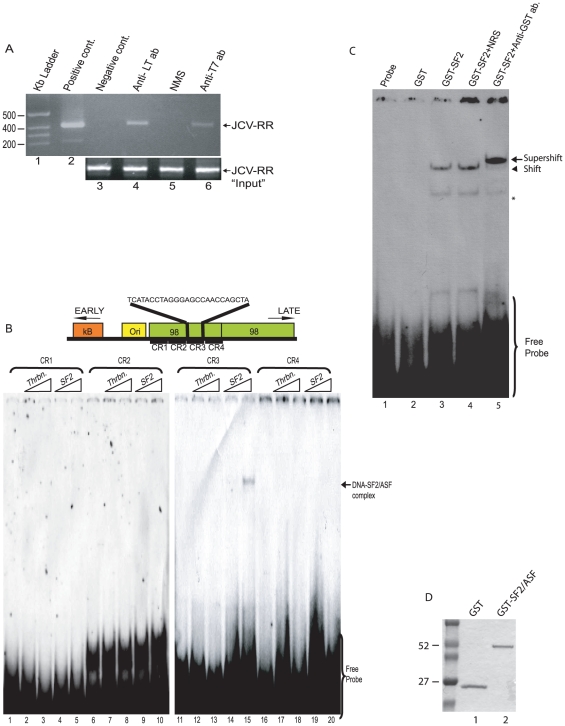
SF2/ASF directly targets JCV regulatory region in vivo and in vitro. A. BSB8 cells were transfected with an T7-SF2/ASF expression vector, cross-linked and ChiP assay performed using antibodies to Large T antigen (lane 4), Normal mouse serum (lane 5), T7 tagged SF2/ASF (lane 6), and no antibody (lane 3). In lane 2, JCV Mad-1 genomic DNA was used as positive control. B. Gel shift analysis of JCV oligonucleotides spanning the 98-bp repeated region of the viral promoter with recombinant SF2/ASF. Four oligonucleotide probes (CR1, CR2, CR3, CR4), and JCV regulatory region with early and late orientation are schematized at the top of the panel. C. Antibody supershift gel electrophoresis. CR3 oligonucleotide and GST-SF2/ASF complexes were incubated with normal rabbit serum (NRS) or anti-GST antibodies for an additional 20 min prior to gel electrophoresis. The star depicts a nonspecific band and the arrowhead points shifted specific complexes and the arrow points antibody supershifted specific complexes. D. Coomassie blue staining of GST (lane 1) and GST-SF2/ASF (lane 2).

To better understand the events associated with SF2/ASF suppression of JCV promoter activity, we designed a series of experiments to assess the ability of SF2/ASF to bind to the JCV promoter sequence. ChIP analysis of BSB8 cells demonstrated association of SF2/ASF with the JCV regulatory region. The DNA corresponding to the 98 bp tandem sequence of the JCV promoter was immunoprecipitated with antibody that pulled down SF2/ASF, suggesting the association of SF2/ASF with JCV DNA in BSB8 cells (Fig. 4A). To more precisely identify the region within the viral promoter that is a binding site for SF2/ASF, we used a series of oligonucleotides, named CR1, CR2, CR3, and CR4, (Fig. 4B), as probes in gel shift assays. To avoid any problems associated with non-specific binding, we used highly purified bacterially produced SF2/ASF in the binding reaction. As shown in Fig. 4B only CR3, which corresponds to the central region of the 98 bp repeat, showed binding activity to SF2/ASF. Furthermore, results from the supershift studies pointed to association of GST-SF2/ASF with the CR3 domain of JCV promoter (Fig.4C).

**Figure 5:**
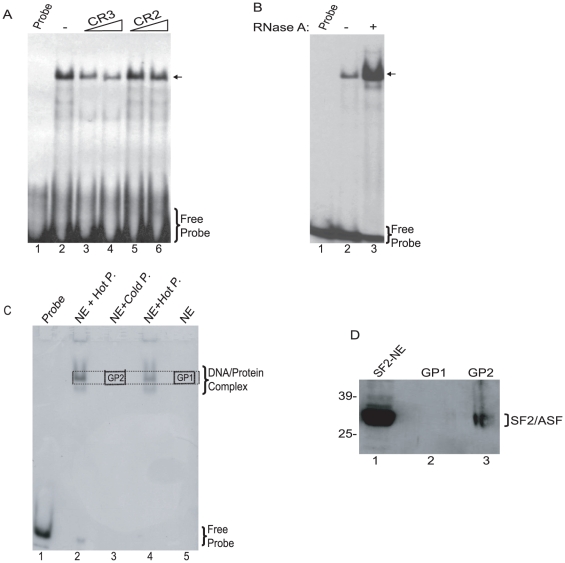
Characterization of the interaction between SF2/ASF and JCV regulatory region by gel shift assays. A. Competition analyses of the CR3 oligonucleotide of JCV regulatory region. Nuclear extracts from PHFA cells incubated with end-labeled double stranded CR3 oligonucleotide probe in the presence of cold CR3 (lanes 3 and 4) and CR2 (lanes 5 and 6) oligonucleotides. B. Presence of RNA in nuclear extracts influences the binding pattern to CR3 oligonucleotide. Nuclear extracts from PHFA cells incubated with end-labeled double stranded CR3 oligonucleotide probe in the presence or absence of RNase A (lanes 2 and 3, respectively). C. Gel shift analysis of JCV CR3 oligonucleotide in the primary human fetal glial cell extracts. End-labeled (lanes 2 and 4) or cold (lane 3) CR3 oligonucleotide incubated with PHFA nuclear extracts. Radioactively-labeled CR3/NP complexes (lanes 2 and 4) were used as reference to label and to cut the gel pieces containing the CR3/NP complexes in cold oligonucleotide reaction (lane 3, GP2) and in nuclear extract control lane (lane 5, GP1). Dotted-lines indicate the vertical and horizontal lining of expected running pattern of CR3/NP complexes. Solid boxes indicate the gel pieces (GP1, GP2), that were cut from the native gel. D. Nucleoprotein complexes in native gel pieces (GP1 and GP2) from panel C were denatured, resolved by SDS-PAGE, and were analyzed by Western blotting, using a specific anti-SF2/ASF antibody. In lane 1, nuclear extract from PHFA cells was loaded as positive control.

Next, we performed a series of gel shift experiments using nuclear extracts from PHFA and CR3 DNA probe to assess SF2/ASF interaction with the JCV DNA sequence. Results from gel shift experiments showed the detection of a major DNA-protein complex whose formation was disturbed by unlabeled CR3 DNA competitor but not by CR2 under similar conditions, pointing to the specificity of the DNA-protein complex (Fig. 5A). The addition of RNase to the binding reaction enhanced the CR3-protein complex, suggesting that removal of the RNA molecules in the extract facilitates the interaction of CR3 binding protein to the CR3 DNA probe. This is an interesting observation as SF2/ASF is a known RNA binding protein. To investigate the identity of the protein associated with CR3, a CR3-associated complex in PHFA was fractionated on a preparative native polyacrylamide gel (Fig. 5C) and the complex was analyzed by SDS-PAGE followed by Western blot using anti-SF2/ASF antibody. As seen in Fig. 5D, a sample corresponding to the CR3 nucleoprotein complex named GP2 reacted with anti-SF2/ASF antibody, indicative of SF2/ASF association with CR3 nucleoprotein complex.

### RRM1 domain of SF2/ASF interacts with the JCV promoter DNA sequence and inhibits viral gene transcription

**Figure 6:**
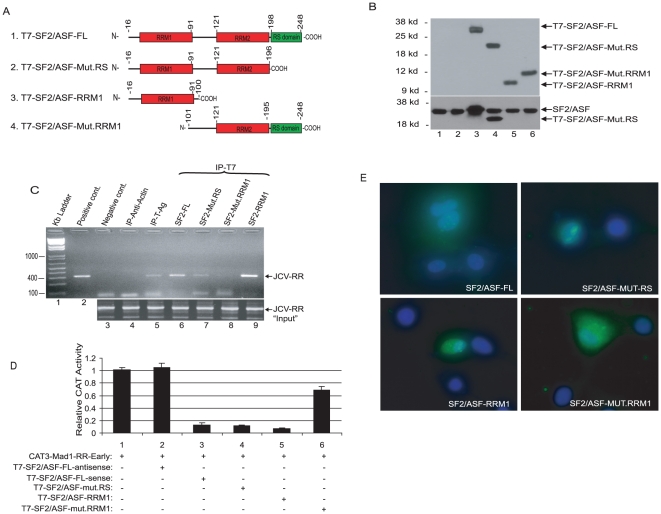
“RRM1” domain of SF2/ASF is required for the inhibition of JCV-Early promoter. A. Schematic representation of SF2/ASF-FL (1) and its functional domain mutations, Mut.RS (2), RRM1-alone (3), and Mut.RRM1 (4). B. Expression of T7-SF2/ASF-FL and truncated forms of SF2/ASF were detected by using anti-T7 (top panel) and anti-SF2/ASF (bottom panel) antibodies. C. BSB8 cells transfected with T7-SF2/ASF constructs, schematized in panel A, cross-linked and ChiP assay performed using antibodies to actin (lane 4), to T-Ag (lane 5), to T7 (lanes 6 to 9), and no antibody (lane 3). D. JCV_E_ reporter plasmid transiently transfected into PHFA cells either alone (lane 1) or in combination with T7-SF2/ASF-FL-antisense (lane 2), T7-SF2/ASF-FL-sense (lane 3), T7-SF2/ASF-Mut.RS (lane 4), T7-SF2/ASF-RRM1 (lane 5), and T7-SF2/ASF-Mut.RRM1 (lane 6) expression plasmids. E. Subcellular localization of SF2/ASF and its truncated forms in PHFA cells.

SF2/ASF has a modular structure consisting of two copies of the N-terminus RNA recognition motif, RRM1 and RRM2, followed by an arginine-serine rich, RS, domain in its C-terminus [16], [21], (Fig. 6A). To assess the importance of these domains in the control of JCV gene transcription, we created a series of deletion mutants of SF2/ASF and examined their expression in PHFA (Fig. 6A and B). The association of each mutant protein with the JCV promoter was investigated by ChIP assay and the results showed that mutant protein with no RRM1 showed no binding activity to JCV DNA. On the other hand, mutant protein containing only RRM1 exhibited strong binding activity to the JCV promoter (Fig. 6C). Results from transcription experiments showed that expression of RRM1 in PHFA is sufficient for suppression of the JCV promoter by SF2/ASF protein (Fig. 6D).

Previous studies have indicated that SF2/ASF mainly localizes to the nucleus in various cell types and it shuttles between the nucleus and cytoplasm [16], [21], [22]. In accordance with this observation, analyses of the sub-cellular localization of SF2/ASF in PHFA cultures by immunocytochemistry also showed nuclear localization with speckled appearance (Fig. 6E). Interestingly, the SF2-Mut-RRM1 construct, which retains RRM2 and RS domains, was localized in both of the cellular compartments, nucleus and cytoplasm. On the other hand, the RRM1 domain alone was exclusively localized to the nucleus in PHFA cells.

### Suppression of SF2/ASF promotes JCV replication in astrocytes

**Figure 7:**
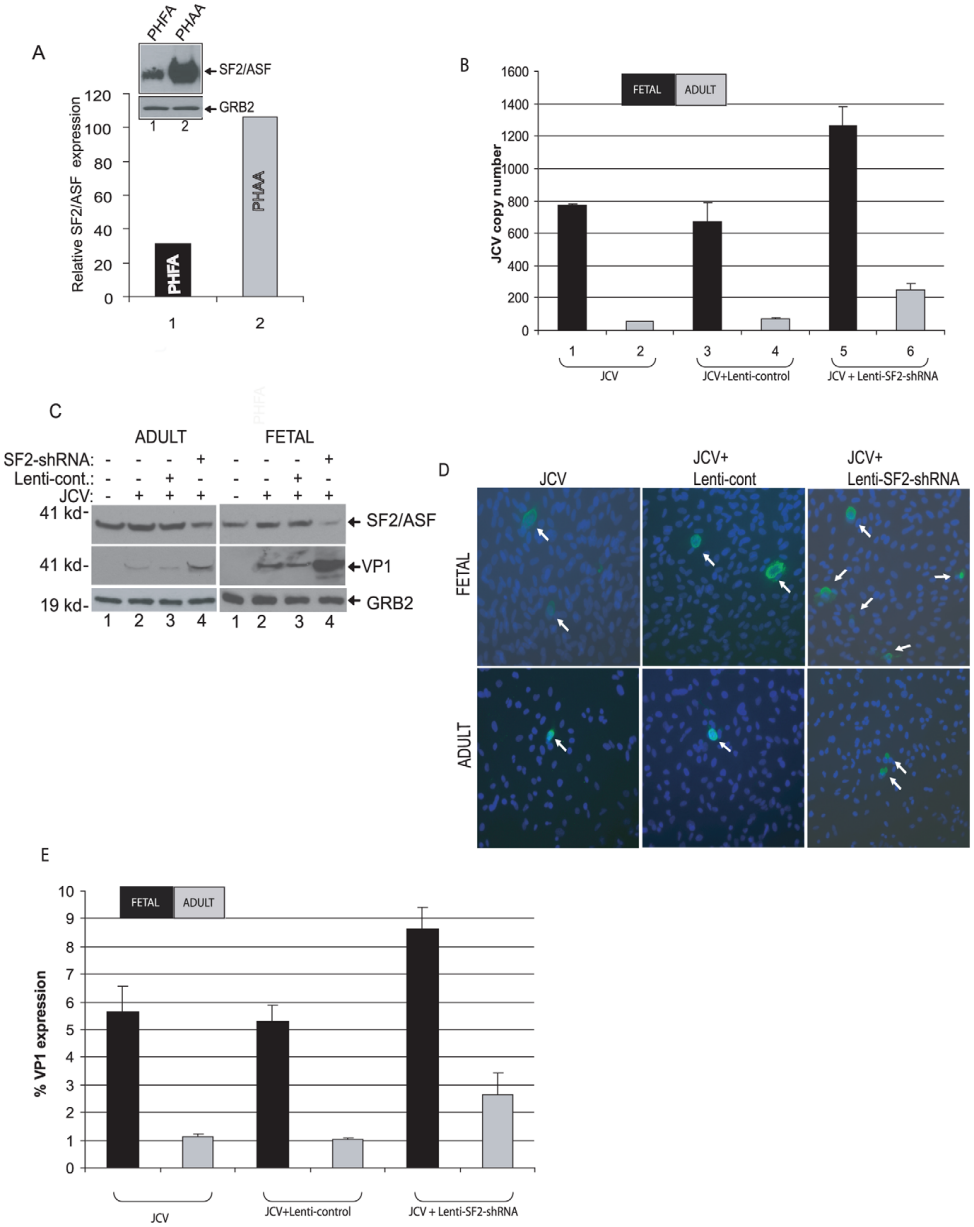
Downregulation of SF2/ASF induces JCV propagation in primary human fetal and adult astrocytes. A. Western blot analyses of SF2/ASF expression in PHFA and PHAA cells. Grb2 was probed as a loading control. Band intensities were quantified and shown as a bar graph. B. PHFA and PHAA cells infected with a lentivirus encoding SF2/ASF-shRNAs at day 0, transfected/infected with JCV Mad-1 strain at day 1. Q-PCR analyses of JCV viral particles in culture supernatants were performed as described in Fig. 1C. C. Whole cells extracts were prepared from the same infections as described in panel B and processed for Western blotting by using specific antibodies against SF2/ASF and VP1. Grb2 was probed as loading control. D. Immunocytochemical analyses of VP1 expression in PHFA and PHAA cells infected with JCV. E. Quantification of VP1 expression in infected cells from panel D.

Examination of SF2/ASF levels in primary fetal and adult astrocytes showed lower levels of this protein in fetal astrocytes compared to those in adult astrocytes, which had more than a three-fold higher level (Fig. 7A). This was an interesting observation as earlier results from our lab showed poor infection by JCV of adult astrocytes compared to fetal astrocytes. To investigate the involvement of SF2/ASF on JCV replication in these cells, we down-regulated expression of SF2/ASF with a lentivirus encoding shRNA that specifically targets SF2/ASF expression (Fig. S4) and demonstrated that the introduction of lenti-SF2/ASF shRNA but not the control lentivirus in the JCV infected cells increased viral production in both fetal and adult astrocytes (Fig. 7B). Results from Western blot analysis verified an increased level of viral capsid protein production, VP1, in cells with a decreased level of SF2/ASF (Fig. 7C). Further, results from immunocytochemical staining of the infected cells showed 53% and 136% increase in the number of VP1 positive cells in fetal and adult infected astrocytes, respectively. Of note, lenti-SF2/ASF shRNA was able to suppress 64% and 72% of SF2/ASF expression in adult and fetal astrocytes, respectively (shown in Fig. 7C). Thus, it is evident that a modest decrease in the level of SF2/ASF can augment the level of JCV replication in the permissive cells. In addition, immunohistological evaluation of brain sections from PML patients showed a marked decrease in the level of SF2/ASF expression when compared to normal brain sections (Fig. S2).

## Discussion

It is believed that JCV may enter many cell types, yet only under certain immunosuppressive conditions does it enter into a lytic infection cycle in glial cells. Earlier studies established that cell type-specific reactivation of JCV in glial cells is primarily regulated at the transcriptional level [19], [20]. In this respect, several transcription factors including YB-1, Purα, GF-1, Egr-1, c-jun, NF1, and others were identified based on their ability to modulate JCV promoter activities in glial cells. Of note, none of these regulators were restricted to glial cells, and their overexpression, in most areas, failed to stimulate transcription of JCV in non-glial cells, suggesting that the JCV promoter in general is under a negative regulation in non-permissive cells. Accordingly, one may speculate on the involvement of inducible glial specific activator which becomes reactivated and promotes JCV gene transcription and replication during the course of immunosuppression. Thus, it is conceivable that the combination of ubiquitous negative and inducible positive glial specific factors determines the level of viral gene expression in glial and non-glial cells. Here, we identified an RNA splicing regulatory protein, SF2/ASF, which is ubiquitously expressed in all cell types as a negative regulator of JCV gene transcription. This is not a universal event for the other members of the polyomavirus family including SV40 and BKV and requires a specific DNA sequence located within the 98 bp tandem promoter region. Our results demonstrate that a decrease in the level of SF2/ASF increases the level of viral gene expression and replication in glial cells and has no positive impact on JCV expression in non-glial cells (Fig. S3), suggesting that a positive glial-specific activator is required to initiate viral gene expression. SF2/ASF also suppresses expression of the JCV promoter in transformed cells whose viral genome is integrated in host chromosomes. Our DNA binding experiments identified a CR3 region of the JCV 98 bp repeat that is conserved in PML (Mad) and non-PML (archetype) strains of JCV as the primary target for SF2/ASF. This region encompasses several predicted binding sites for DNA binding proteins including Purα, NF-1, MF3, Elk-1, COE1, p300, and Zic3. At present, the importance of these transcription regulators and their potential interplay with SF2/ASF remains to be investigated.

SF2/ASF exhibits a modular structure consisting of two copies of RNA binding motifs (RRM1 and RRM2), followed by an arginine-serine rich (RS) domain [21]. Whereas the RRM domains are responsible for RNA binding activity of ASF/SF2, the RS region interacts with the core-splicing components [15], [16]. Our results showed that the RRM1 domain has the capacity to interact with the DNA sequence of JCV and that this interaction is important for suppression of viral gene transcription.

Examination of SF2/ASF levels in several glial-derived cells including human astrocytic tumor cells, U-87 MG, primary human fetal astrocytes (PHFA) and primary human adult astrocytes (PHAA) showed higher levels of SF2/ASF expression in U-87 MG and PHAA where virus replicates poorly in comparison to PHFA, routinely utilized for virus propagation. Our results demonstrated that a decrease in the level of SF2/ASF in both PHFA and PHAA increases the level of viral replication. Altogether, results from the studies presented here identified a novel regulatory pathway involving splicing factor SF2/ASF in suppression of JCV gene transcription. This is a first report on the ability of SF2/ASF to control viral gene expression at the transcriptional level and suggests the operation of a novel regulatory event that controls reactivation of JCV in non-glial as well as glial cells.

## Materials and Methods

### Plasmid Constructs

pCGT7-SF2/ASF-FL expression plasmid, kindly provided by Javier F. Cáceres (Medical Research Council Human Genetics Unit, Western General Hospital, Edinburgh EH4 2XU, Scotland, United Kingdom) and was described previously [22]. pCGT7-SF2/ASF-antisense expression plasmid was created with the following primers: SF2 -F: 5′-ACCTTCCAGGATCCATGTCGGGAGGTGGTGTGATT-3 and SF2-R: 5′-ACCTTCCATCTAGATTATGTACGAGAGCGAGATCT-3′, and was cloned into the pCGT7 vector in reverse orientation at XbaI/BamH1 sites. SF2/ASF domain mutations were created by using the following primers: SF2-Mut.RS (1–196)-forward 5′-ACCTTCCATCTAGATCGGGAGGTGGTG TGATTCGT-3′, SF2-Mut.RS (1–196)-reverse: 5′-ACCTTCCAGGATCCTTACCCATCA ACTTTAACCCGG-3′; SF2-RRM1 (1–100)-forward: 5′-ACCTTCCATCTAGATCGGGAG GTGGTGTGATTCGT-3′, SF2-RRM1 (1–100)-reverse: 5′-ACCTTCCAGGATCCTTA GCCGCCGCCTCGGCCTGT -3′. SF2-Mut.RRM1 (101–248)-forward: 5′-ACCTTCCATCTAGAGGCGGGGGTG GAGGTGGCGGA-3′, SF2-Mut.RRM1-(101–248) - reverse: 5′-ACCTTCCAGGATCCTTA TGTACGAGAGCGAGATCTGCT-3′. pcDNA 3.1 (+) JCV-Early expression plasmid was described earlier [23]. pBluescript-JCV-Early construct was created as follows. JCV early genome was amplified by using the following primers: Mad-1F (248–225), 5′-CTGGCTCGCAAAACATGTTCCCTT– 3′; Mad-1R (2575-2601), 5′-TATAACCAGCTTTACTTAACAGTTGCA-3′. The amplification construct was cloned into pBlue-Script KS plasmid.

### Cell cultures

Human malignant glioma cell line, U-87 MG, was obtained from American Type Culture Collection (ATCC), and was grown in Dulbecco's Modified Eagle's Medium (DMEM) containing 10% heat-inactivated fetal bovine serum (FBS) and penicillin/streptomycin (100 µg/ml). Primary human fetal glial cells were prepared as previously described [24]. BSB8 cells were derived from cerebellar medulloblastomas that developed in transgenic mice expressing the early genome of the JCV [25]. SVG-A is a human glial cell line which was established by transformation of primary human fetal glial cells with an origin-defective SV40 virus [26]. All cell lines and cultures were maintained at 37°C in a humidified atmosphere with 7% CO_2_.

### JCV infection

Transfection/infection of cells with the full-length JCV Mad-1 genome was described previously [27], [28]. Briefly, PHFA cells, at a confluence of 1×10^6^ cells per T75-cm tissue culture flask, were co-transfected/infected with the full-length JCV DNA (10 µg/flask) in the presence or absence of pCGT7 plasmid expressing SF2/ASF in the sense or antisense orientation using Fugene6 transfection as indicated by the manufacturer (Roche). The total amount of DNA in each transfection mixture was kept constant (14 µg) by the addition of empty pCGT7 vector. At 8 days post-infection, cells were trypsinized and split into two equal portions. One half was used for preparation of whole cell protein extract for Western blot analysis, and the other half was split into two equal volumes for DNA and RNA preparation using Qiaprep Spin Miniprep kit (Qiagen), and RNeasy Mini kit (Qiagen), respectively.

### DpnI assay and detection of replicated-viral DNA by Southern blotting

Low molecular weight DNA purified from JCV-infected cells was digested with Dpn I and BamH1 enzymes. Digested-DNA samples were separated on 1% agarose gel and were transferred to a nylon membrane. Replicated viral DNA was visualized upon incubation of the membrane with a [^32^P]-labeled JCV DNA probe as described earlier [28].

### RT-PCR

Total cellular RNA was extracted from JCV-infected PHFA cells by using the Qiagen RNeasy kit according to the manufacturer's recommendations. After treatment with DNase I, followed by phenol/chloroform extraction and ethanol precipitation, cDNAs were synthesized using M-MuLV reverse transcriptase. RNA templates were removed by RNase H digestion. A total of 100 ng cDNA was used as template for PCR reactions. Viral transcripts from JCV infected cells were determined by using following primers in RT-PCR reactions. VP1-forward: 5′-CTCATGTGGGAGGCTGTGAC-3′, VP1-reverse: 5′- TCCTCCTGTTAGTGTCCCAA-3, Large T-forward: 5′-ATGGACAAAGTG CTGAATAGG-3′, Large T-reverse: 5′-TAGTGGTATACACAGCAAAAG-3′ and GAPDH- Forward: 5′- TTCTCCCCATTCCGTCTTCC -3′, GAPDH-reverse: 5′-GTACATGGTATT CACCACCC-3′. To analyze the effect of SF2/ASF on JCV early gene expression, PHFA and BSB8 cells were transfected with pCGT7-control or with pCGT7-SF2/ASF expression plasmids. At 48 h of post-transfection, cells were harvested, and total RNA was extracted with an RNA extraction kit (RNeasy, Qiagen) according to the manufacturer's instructions. One microgram of RNA was then reverse-transcribed using M-MuLV-reverse transcriptase and oligo-dT primers. JCV Mad-1 genomic DNA and U87 MG cells were used as positive and negative controls of amplifications, respectively. RT-PCR reactions of the JCV-early region splicing were performed by using following primers: PF (Mad-1 4801-4780): 5′- CCTGATTTTGGTACATGGAA -3′ and PR (Mad-1 4291- 4313): 5′-GTGGGGTAGAGTGTTGGGATCCT -3′. Amplified gene products were resolved on a 3% DNA-agarose gel.

### Detection of viral particles in culture medium by Q-PCR

Transfection/infection of cells with the full-length JCV-Mad1 genome was performed as described above. The culture medium (containing viral particles) was collected at 8 days post-infection, and after centrifugation at 10,000 rpm for 10 minutes to remove cell debris, supernatants were collected and incubated at 95°C for 10 minutes to inactivate virus. Ten microliters of the medium was then used as a template in Q-PCR reactions. The standard curve was obtained after serial dilution of pJCV, a plasmid containing the whole genome of the JCV Mad-1 strain, knowing that 10 ng of pJCV correspond to 109 copies of viral genome. The standard curve was then used to extrapolate the viral load of each sample. Negative and positive controls were included in each reaction and each sample was tested in triplicate. All Q-PCR analyses were done by using Lightcycler 480 (Roche). Primers were JCV Q-PCR-forward: 5′-AGTTGATGGGCAGCCTATGTA-3′ and JCV Q-PCR-reverse: 5′- TCATGTCTGGGTCCCCTGGA-3′. The probe for the Q-PCR was 5′-/5HEX/CATGGA TGCTCAAGTAGAGGAGGTTAGAGTTT/3BHQ_1/-3′.

### Reporter gene assays

Reporter gene constructs containing the regulatory region of the JCV Mad-1 strain was described previously [23]. Briefly, Mad-1 (4989 to 480) region was PCR-amplified and inserted into the BamHI site of the pBLCAT_3_ vector in early and late orientations. The resulting plasmids were called pBLCAT_3_-Mad1-Early and pBLCAT3-Mad1-Late. pBLCAT3-BKV-Early and pBLCAT3-BKV-Late plasmids were described previously [29]. PHFA cells were transfected with these constructs in the presence or absence of expression plasmids for SF2/ASF-FL and its mutant forms. At 48h post-transfection, cells were extracted with a series of freeze/thaw cycles, and the CAT activity of the samples was determined.

### Immunocytochemistry

In order to show localization of SF2/ASF in glial cells, PHFA cells were seeded in two-well chamber slides and transfected with pCGT7-SF2-FL, pCGT7-SF2-Mut.RS, pCGT7-SF2-Mut.RRM1, and pCGT7-SF2-RRM1 plasmids. After 48 h, cells were fixed with cold acetone/methanol (1/1) for 2 minutes and washed three times with PBS. Cells were treated with a 5% BSA solution, followed by incubation with T7 monoclonal antibody. Cells were then incubated with FITC-conjugated secondary antibody, mounted with aqueous mounting medium, and examined under immunoflourescence microscope. To analyze infection of PHAA and PHFA with JCV, cells were plated in 2 well chamber slides at 10 day post-infection and fixed with cold acetone/methanol (1/1). After treatment with a 5% BSA solution, samples were incubated with JCV VP1-specific monoclonal antibody followed by incubation with FITC-conjugated secondary antibody. Samples were mounted with aqueous mounting with DAPI, and examined for microscopic immunoflourescence.

### Production and purification of GST proteins

SF2/ASF was amplified with the following primers, GST-SF2-Forward: 5′-ACCTTCCAGGATCCATGTCGGGAGGTG GTGTGATT-3′, GST-SF2-Reverse: 5′-ACCTTCCAGAATTCTTATGTACGAGAG CGAGATCT -3′, and cloned into BamHI and EcoRI sites of the pGEX2T E-coli expression vector. Bacterial cultures were incubated in one liter LB growth medium at 37°C until they reached a conflucence of 0.6 at 595.0 nm wavelength (wl) after which they were transferred to 28°C and treated with IPTG (5 µg/ml). Cultures were centrifuged at 5 k rpm for 10 min and re-suspended in 20 ml of PENT buffer (20 mM tris-HCL pH 8.0, 100 mM NaCl, 1 mM EDTA, 0.5% NP-40 1% N-L-sarcosyl, and general protease inhibitors), after which they were lyzed by sonication. Bacterial lysates were centrifuged at 15 k rpm for 15 minutes. Clear lysates were incubated with 150 µl of glutathione sepharose beads overnight, centrifuged at 3 k rpm for 5 minutes, and resuspended in TNN buffer (150 mM NaCl, 40 mM Tris pH 7.4, 1% NP-40 (ipegal), 1 mM DTT, 1 mM EDTA). Beads were washed in TNN buffer three times, and resuspended in PBS. The integrity of the proteins was tested by SDS-PAGE. SF2/ASF-FL protein was cleaved from the GST fusion protein by thrombin, and was subsequently used in gel shift experiments.

### Chromatin immunoprecipitation, (ChIP) assay

BSB8 cells were transiently transfected with pCGT7-SF2/ASF full length and its truncated forms. ChIP assays were performed as described previously [30]. Briefly, proteins were cross-linked to DNA by formaldehyde, following by sonication to fragment the chromatin and immunoprecipitation of specific proteins to obtain DNA segments. Cross-linking was reversed and DNA was analyzed by PCR.

### RNA interference

To knock-down expression of SF2/ASF, PHFA cells were seeded (5×10^5^ cells per well) in 60 mm dishes. After 24 h, cells were transfected with 200 pmol short interfering RNA (siRNA) per well (Santa Cruz), using Oligofectamine (Invitrogen). After 72 h, total proteins were prepared. Lentivirus-based U6-promoted SF2 shRNA constructs were generated by cloning PCR products carrying sense and antisense oligonucleotides of SF2/ASF into the pLL3.7 vector as described previously [31]. Two SF2 shRNA constructs were made to target the nucleotide sequences 35–55 (shA), and 423-443 (shB) of the human SF2 cDNA (GenBank accession number AAH33785.1). The viruses were packaged in 293T (human embryonic kidney) cells according to the procedure described previously [32]. Primary human fetal and adult astrocytes were plated in six-well plates at 50% confluency and were incubated with 1 ml of the viral supernatants. The infected cells were then kept in regular complete medium for 48 h. Inspection with fluorescence microscopy confirmed the presence of more than 80% of GFP-positive cells after viral infection. The level of SF2 in infected cells was evaluated by Western blot analysis.

### Immunohisthochemistry

Immunohisthochemistry was performed using the Avidin-Biotin-Peroxidase method according to manufacturer's suggestions (Vector Laboratories). Control and PML brain sections were blocked with 5% normal horse serum in 0.1% PBS/BSA for 2 h at room temperature. SF2/ASF antibody (Clone 96, 1∶500 dilution, Invitrogen) was incubated overnight at room temperature. Biotinylated secondary anti-mouse was incubated for 1 h at room temperature (1∶250 dilution). Sections were incubated with avidin-biotin (Vector Laboratories) for 1 h at room temperature, rinsed with PBS, and developed with deaminobenzidine (Sigma). Finally, the sections were mounted with Permount (Fisher Scientific).

### Gel shift assay

The following oligonucleotides were used in band shift assays: κB: 5′-AAAACAAGGGAATTTCCCTGGCCTC-3′. ORI: 5′-GGAGGCGGAGGCGGCCTCGGCC TCC -3′. CR1: 5′-CCTGTATATATAAAAAAAAGGGAAG-3′. CR2: 5′-GGATAGCTGCCA GC CAACGATGAGC-3′. CR3: 5′-TCATACCTAGGGAGCCAACCAGCTA-3′. CR4: 5′-AC AGCCAGTAAACAAAGCACAAGGC-3′. Band shift assays were carried out as described previously [33], [34]. Recombinant SF2 or nuclear extracts from PHFA were incubated with [^32^P]-end labeled oligonucleotides (50,000 cpm/reaction) in a binding buffer containing 1.0 µg poly (dI–dC), 12 mM HEPES (pH 7.9), 4 mM Trish [pH 7.5], 60 mM KCl, 5 mM MgCl_2_ and 1.0 mM DTT. The reaction mixture was incubated at 4°C for 1 h to allow assembly of DNA–protein complexes. The complexes were then resolved on a 6% polyacrylamide gel in 0.5× TBE (1× TBE is 89 mM Tris– HCl [pH 8.0], 89 mM boric acid and 2 mM EDTA [pH 8.0]). The gels were dried, and complexes were detected by autoradiography. The presence of SF2/ASF in the CR3/nucleoprotein (CR3/NP) was analyzed by gel shift followed by Western blot analysis. End-labeled or unlabeled CR3 oligonucleotide was incubated with nuclear extracts from PHFA cells and complexes were resolved on 6% native PAGE. End-labeled CR3/NP complexes were used as reference to unlabeled-CR3/NP complexes, and the DNA/protein complexes were cut from the gels and elution of its contents were analyzed by SDS-PAGE followed by Western blot analysis using anti-SF2/ASF antibody.

### MTT (3-(4,5-Dimethylthiazol-2-yl)-2,5-diphenyltetrazolium bromide) assay for cell proliferation

PHFA and PHAA cells were plated in 6-well plates (2×10^5^), and infected either with Lenti-control or Lenti-SF2/ASF-shRNA. Seventy two hours postinfection, cells were washed with PBS, and incubated with 1 ml of MTT working solution (DMEM with 0.5 mg/ml MTT) for 1 hour at 37°C. At the end of the incubatuion, the converted dye was solubilized with 1 ml acidic isopropanol (0.004 M HCL in isopropanol). Suspension was pipetted up and down to make sure that the converted dye dissolved. The dye solution was transferred into 1.5 ml eppendorf tubes, and centrifuged at 15,000 rpm for 5 min. The supernatant was transferred into new eppendorf tube. Absorbance of the converted dye was measured at a wavelength of 570 nm with background subtraction at 650 nm.

## Supporting Information

Figure S1 Effect of SF2/ASF on transcriptional activity of BKV and E2F promoter sequences. PHFA cells were cotransfected with either pCGT7-empty plasmid or pCGT7-SF2/ASF and pBLCAT3-early, pBLCAT3-late, and pBLCAT3-E2F reporter plasmids. CAT enzyme activities were analyzed at 48 hours posttransfection.
(1.31 MB EPS)

Figure S2 SF2/ASF expression in normal and PML brain. Immunohistochemistry for the SF2/ASF was performed as described in materials and methods. Expression of SF2/ASF was detected in the nuclei of the cells in the normal (top panel) and PML (bottom panel) brain sections. A noticeable downregulation of SF2/ASF expression was observed in PML brain sections.
(5.89 MB EPS)

Figure S3 JCV replication in glial and non-glial cells. Primary human fetal astrocytes (PHFA), peripheral blood mononuclear cells (PBMC), human glioblastoma cell line (U87 MG), human ovarian cancer cell line (HeLa), and human B-cell lymphoma cell line (WIL2N) were infected with either Lenti-control or Lenti-SF2/ASF.shRNA. At 48 hours postinfections, the cells were infected with JCV Mad1 strain (30 MOI/cell). JCV genomic DNA was analyzed in growth medium by Q-PCR at 14 days postinfection.
(1.34 MB EPS)

Figure S4 Effect of Lenti-control and Lenti-SF2/ASF-shRNA on the growth of PHFA and PHAA cells. A. PHFA and PHAA cells were plated in 6-well dishes, either infected with Lenti-control and Lenti-SF2/ASF-shRNA or left uninfected. At 72 hours postinfection, an MTT assay was performed to analyze cell viability as described in the "Materials and Methods" section. B. Western blot analyses of SF2/ASF expression in PHFA and PHAA cells infected with Lenti-control and Lenti-SF2/ASF-shRNA at 72 hours postinfection. Grb2 was probed in the same blots as the loading control.
(1.41 MB EPS)
